# HIV, Intussusception, and a Hidden Malignancy Masquerading Abdominal Tuberculosis: Diffuse Large B-Cell Lymphoma Unveiled

**DOI:** 10.7759/cureus.83072

**Published:** 2025-04-27

**Authors:** Mitali Singh, Vaibhav Mane

**Affiliations:** 1 Pathology, Bharati Vidyapeeth (Deemed to be University) Medical College and Hospital, Sangli, IND

**Keywords:** bcl6, cd10 expression, cd20+, diffuse large b cell lymphoma (dlbcl), disseminated tuberculosis, hiv lymphoma, intussusception

## Abstract

Diffuse large B-cell lymphoma (DLBCL) is a common aggressive non-Hodgkin lymphoma, frequently seen in immunocompromised individuals. This report details the case of a 40-year-old HIV-positive female admitted for abdominal pain, vomiting, and imaging suggestive of disseminated tuberculosis, who was ultimately diagnosed with DLBCL of the ileum complicated by intussusception. Initial investigations revealed anemia and thrombocytosis. Contrast-enhanced CT showed multiple necrotic granulomatous lesions and jejunoileal intussusception, mimicking tuberculosis. Surgical exploration and resection of the intussuscepted ileal segment revealed a polypoidal tumor. Histopathological examination indicated a diffuse proliferation of lymphoma cells with plasmacytoid morphology. Immunohistochemistry confirmed DLBCL (germinal center B-cell subtype), with tumor cells positive for Oct-2, CD20, PAX5, BCL-6, and CD10 and negative for CD38, CD138, and MUM1. This case highlights the diagnostic challenges in differentiating intestinal DLBCL from infectious and inflammatory conditions such as tuberculosis in HIV-positive patients. It underscores the commentative role of biopsy and immunohistochemistry in accurate diagnosis and timely initiation of appropriate therapy, ultimately improving patient outcomes.

## Introduction

Diffuse large B-cell lymphoma (DLBCL) is the most common and aggressive subtype of non-Hodgkin lymphoma (NHL), accounting for a significant proportion (around 30-40%) of adult lymphoma cases. It often presents as an extranodal disease, with the gastrointestinal (GI) tract being one of the most frequently involved sites [1,2]. Among immunocompromised individuals, particularly those with HIV/AIDS, the risk of developing DLBCL is markedly increased (45%) due to chronic immune activation and reduced tumor surveillance [3].

The clinical presentation of intestinal DLBCL is often nonspecific, including abdominal pain, weight loss, altered bowel habits, and bowel obstruction [4], making it difficult to distinguish it from other conditions such as intestinal tuberculosis (TB), inflammatory bowel disease, or other malignancies. In endemic regions, TB remains a major differential diagnosis, especially when imaging shows ileocecal involvement, mesenteric lymphadenopathy, and bowel wall thickening. This diagnostic overlap can lead to delays in appropriate management.

Additionally, intussusception, a condition where one segment of the intestine telescopes into another, is a rare but significant manifestation of intestinal lymphomas in adults [5]. While commonly associated with benign causes in pediatric patients, adult intussusception is often secondary to an underlying malignancy, including lymphoproliferative disorders.

Given the histological heterogeneity of lymphomas, accurate diagnosis relies on histopathological examination and immunohistochemistry (IHC). IHC markers such as CD20, CD10, BCL6, MUM1, BCL2, and MIB-1 play a crucial role in differentiating DLBCL from other B-cell lymphomas [6] and distinguishing it from conditions such as plasmablastic lymphoma (PBL) or Burkitt lymphoma, which are also seen in HIV-associated lymphoproliferative disorders. The MIB-1 proliferation index further aids in assessing tumor aggressiveness and prognosis.

This case was chosen for reporting due to its atypical presentation with intussusception and initial suspicion of TB, highlighting the diagnostic challenge of distinguishing intestinal DLBCL from infectious and inflammatory conditions in immunocompromised individuals. By emphasizing the crucial role of biopsy and IHC, this report underscores the importance of early and accurate diagnosis to ensure timely initiation of appropriate therapy, ultimately improving patient outcomes.

## Case presentation

A 40-year-old female, diagnosed with HIV and on antiretroviral therapy for the past month and with a CD4+ T-cell count of 180 cells/mm³ and an HIV viral load of 5,500 copies/mL at the time of presentation, presented with complaints of intermittent abdominal pain and recurrent vomiting for one month. She had no other known comorbidities and was admitted to the Surgery Department of our institute for further evaluation.

Initial laboratory investigations revealed normocytic normochromic anemia with a hemoglobin level of 8.0 g/dL (normal range: 12-16 g/dL) and thrombocytosis with a platelet count of 473,000/cumm (normal range: 150,000-400,000/cumm). The white blood cell count (10,400 cells/cumm) and lymphocyte count (28%) were within normal limits. Further investigations did not indicate iron deficiency as the primary cause. Abdominal ultrasonography demonstrated a significant edematous lesion involving the omentum and mesentery, resulting in bowel loop collapse and ascites. A subsequent contrast-enhanced CT (CECT) scan revealed multiple necrotic granulomatous lesions/abscesses at the level of the liver, pancreas, and right kidney. Additionally, it showed intussusception at the jejunoileal junction, with marked thickening and narrowing of the ileal wall, along with gastric wall thickening, raising suspicion of disseminated TB. Given the patient’s HIV status and the high TB burden in the region, TB was the primary differential. However, sputum acid-fast bacilli (AFB) staining and CBNAAT testing were negative.

**Figure 1:**
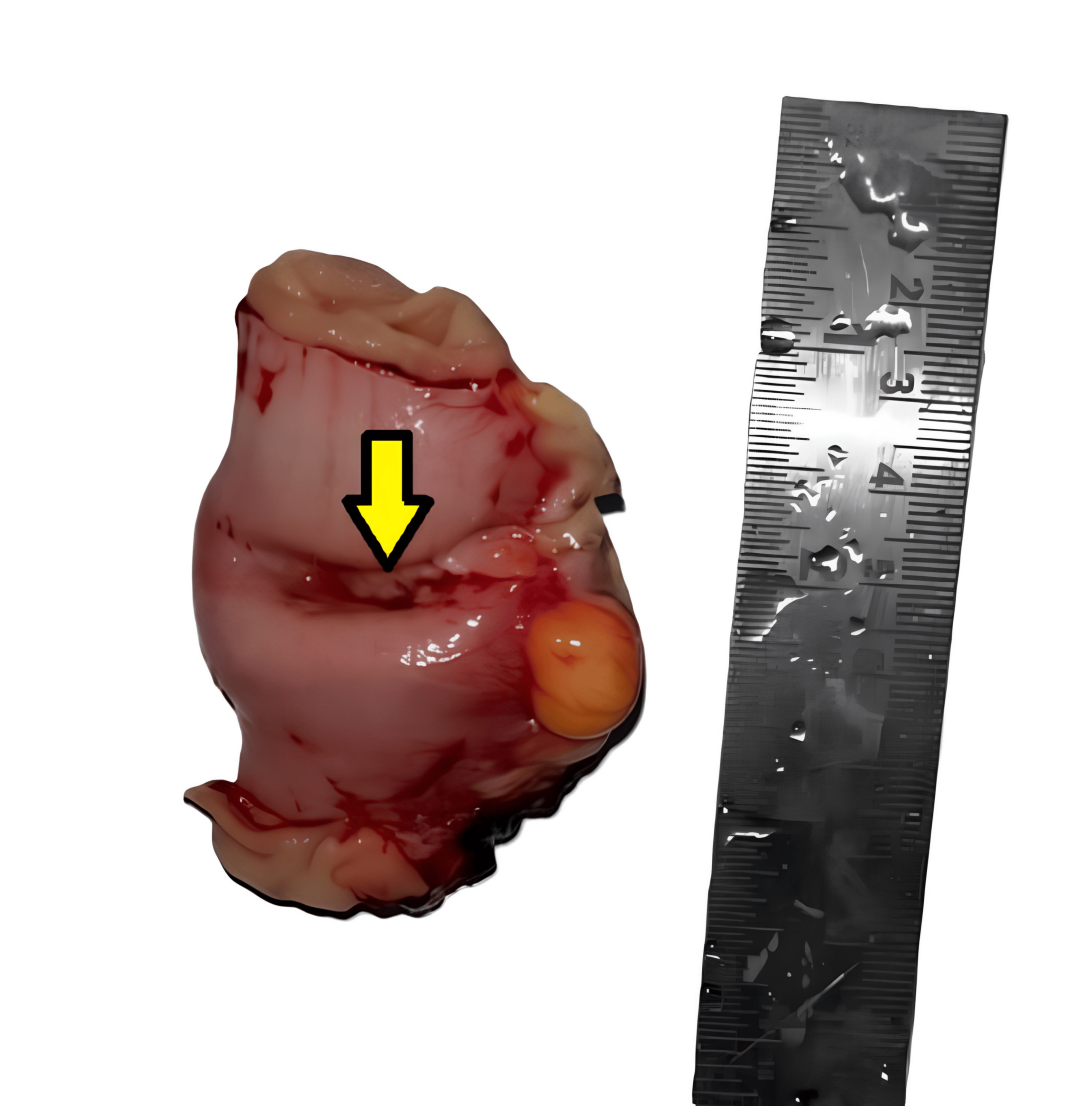
Gross image of the resected ileal segment showing a strictured area.

**Figure 2:**
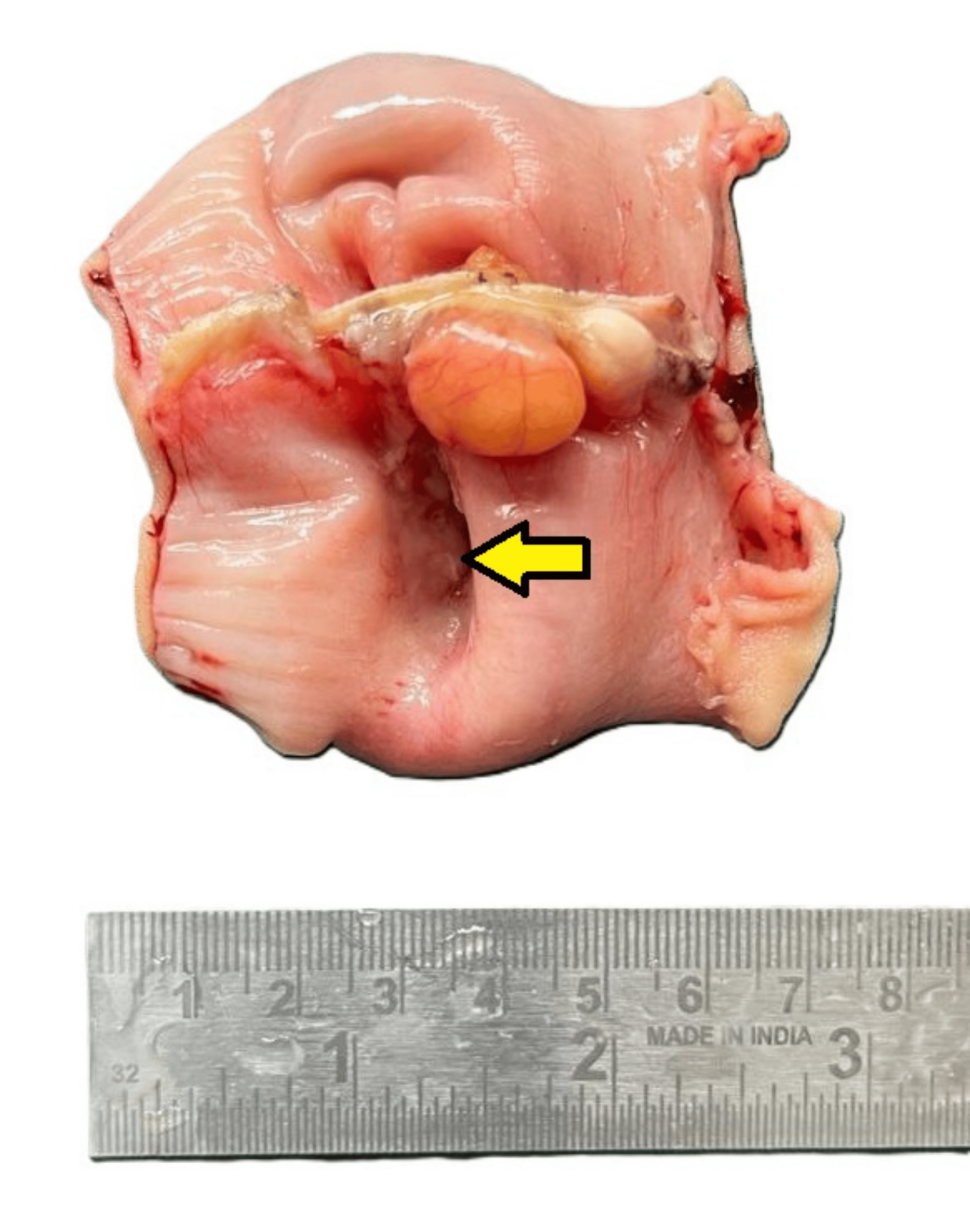
Gross image showing the external surface with focal exudation.

**Figure 3:**
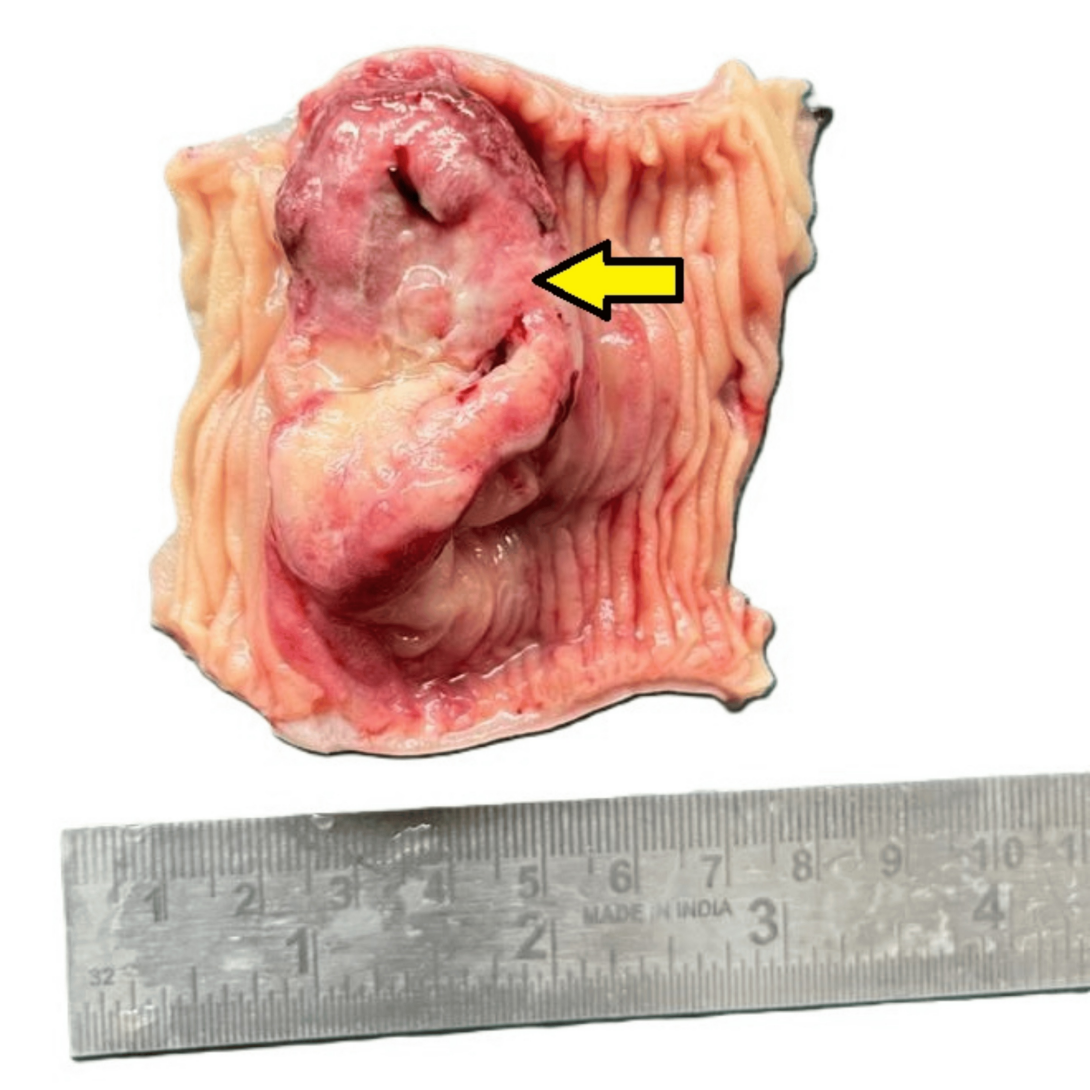
Gross image showing a polypoidal tumor with ulceration.

The patient underwent a planned surgical exploration based on clinicoradiological findings, which revealed jejunoileal intussusception. No significant lymphadenopathy was noted during the intraoperative assessment. The resected ileal segment, measuring 8.5 cm in length, was sent for histopathological examination. Gross examination showed a strictured area focally covered with exudate measuring 3.5 cm in length (Figures 1, 2). Upon opening along the antimesenteric border, a polypoidal tumor with ulceration measuring 5 × 3 × 2 cm was identified, located 2.5 cm and 1 cm from the proximal and distal resection margins, respectively (Figure 3). The cut surface of the tumor appeared greyish-white and involved the full thickness of the intestinal wall up to the serosa. No grossly identifiable lymph nodes were present.

**Figure 4:**
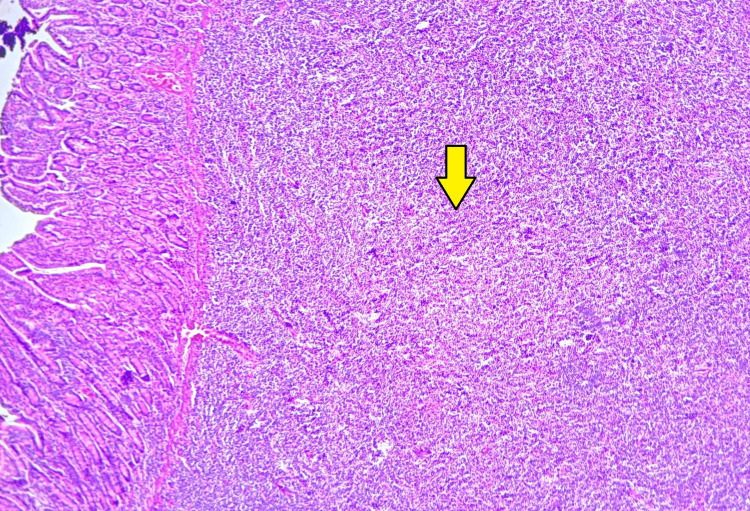
Microscopic image of H&E-stained section showing a submucosal tumor with diffuse proliferation (2x magnification).

**Figure 5:**
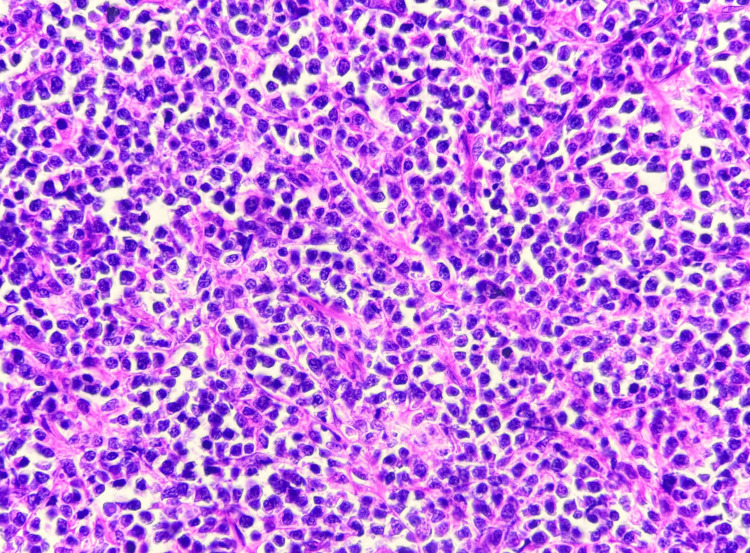
Microscopic image of H&E-stained section showing atypical lymphoplasmacytic cells exhibiting a high nuclear-cytoplasmic ratio, coarse vesicular nuclei, prominent nucleoli, and scant-to-moderate cytoplasm (40x magnification).

Histopathological analysis revealed a submucosal tumor infiltrating through the intestinal wall up to the serosa. Microscopy demonstrated a diffuse proliferation of lymphoma cells with a predominant plasmacytoid morphology. These atypical lymphoplasmacytic cells exhibited a high nuclear-cytoplasmic ratio, coarse vesicular nuclei, prominent nucleoli, and scant-to-moderate cytoplasm. Numerous binucleate cells, atypical mitotic figures, and apoptotic bodies were also observed (Figures 4, 5). Tumor infiltration extended into the mesentery. Based on these findings, two differential diagnoses were considered: DLBCL and PBL.

**Figure 6:**
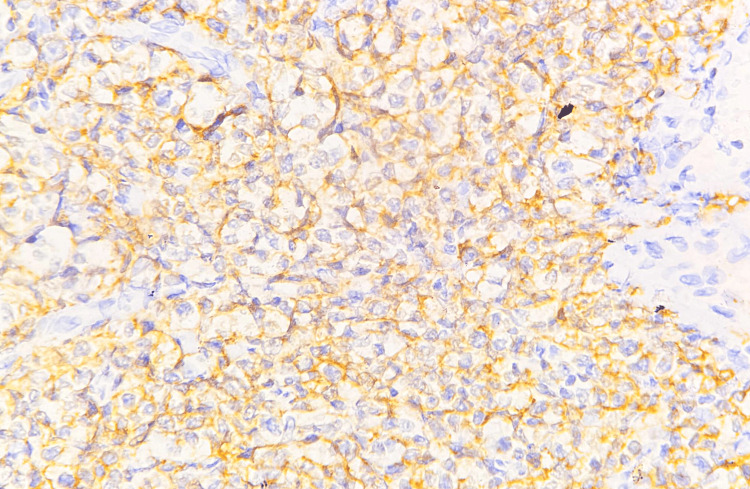
Microscopic image of IHC-stained section showing tumor cells expressing membranous staining for CD20 marker (40x magnification).

**Figure 7:**
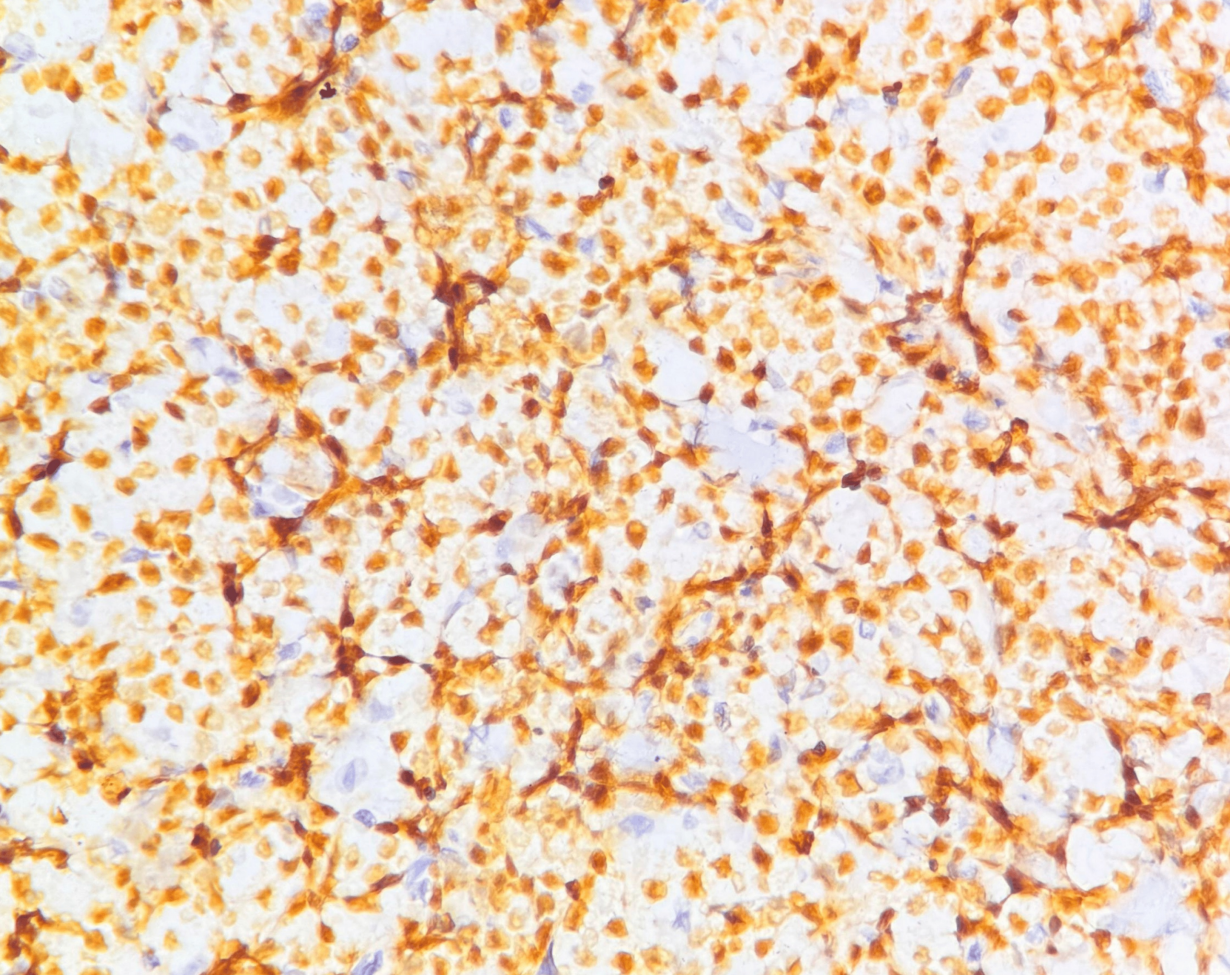
Microscopic image of IHC-stained section showing tumor cells expressing nuclear staining for PAX5 marker (40x magnification).

**Figure 8:**
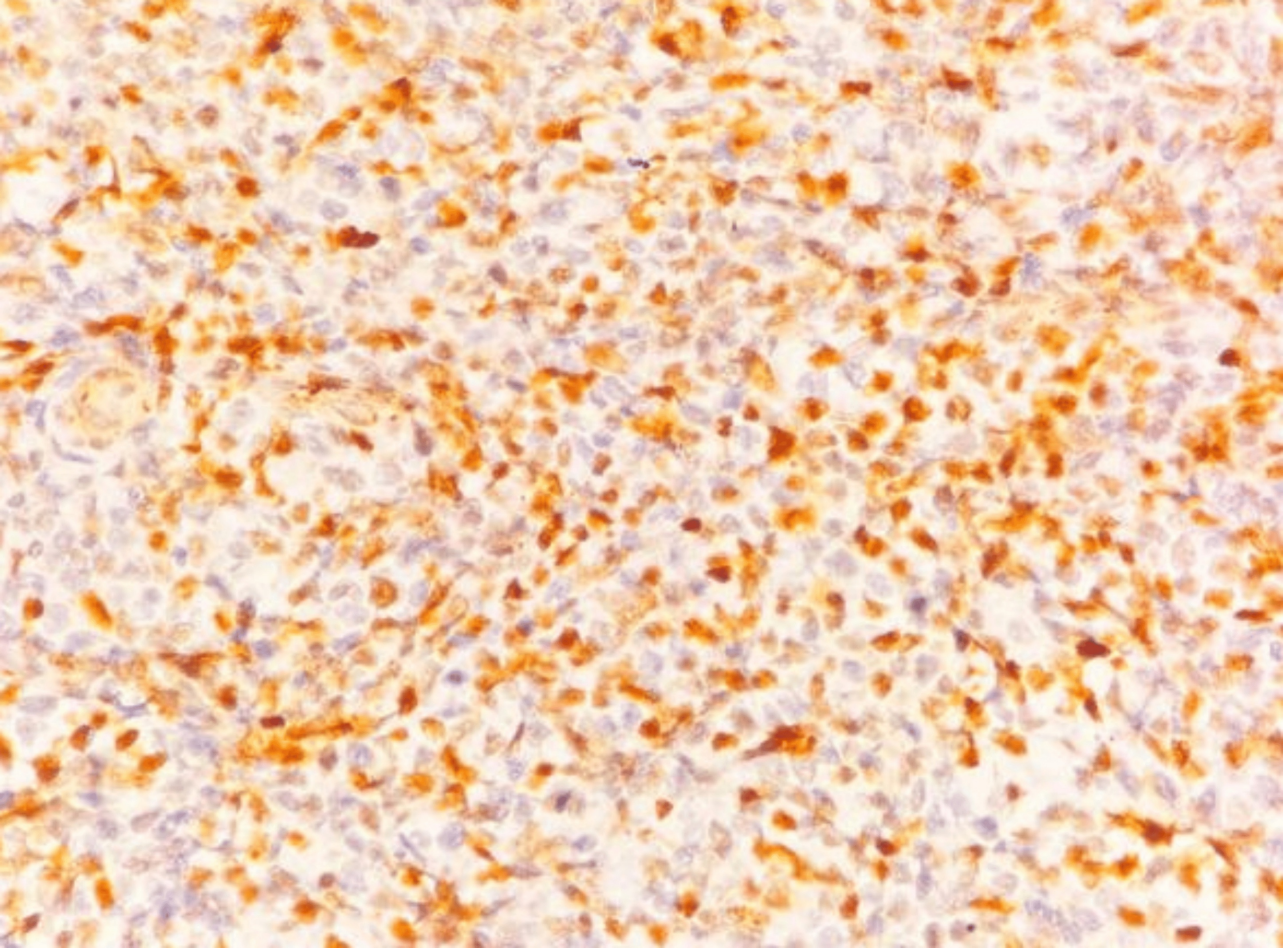
Microscopic image of IHC-stained section showing tumor cells expressing nuclear staining for BCL6 marker (40x magnification).

**Figure 9:**
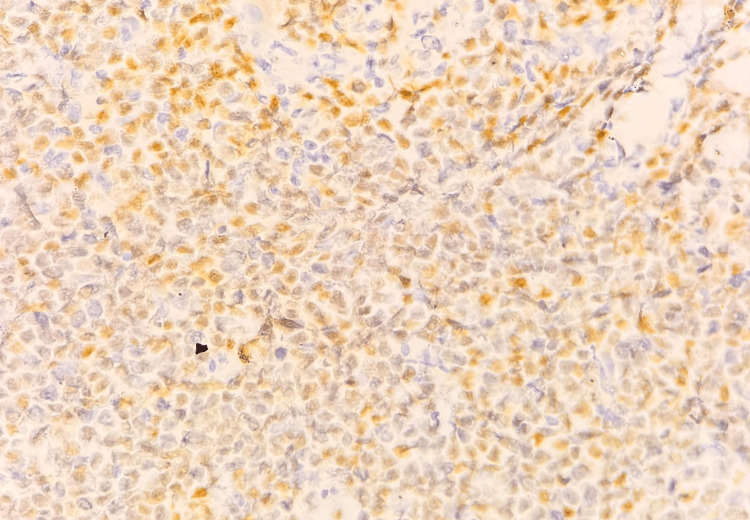
Microscopic image of IHC-stained section showing tumor cells expressing nuclear staining for CD10 marker (40x magnification).

**Figure 10:**
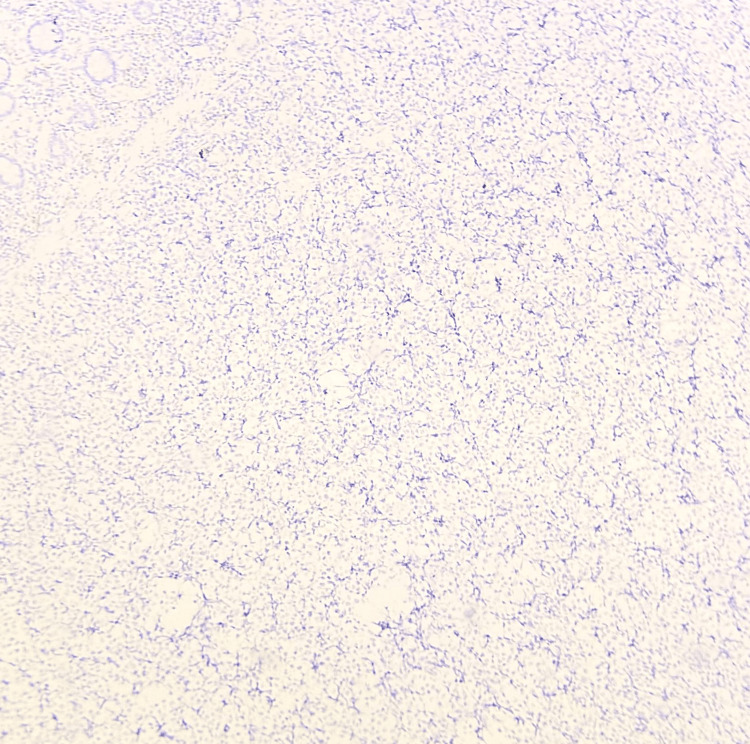
Microscopic image of IHC-stained section showing tumor cells with negative staining for CD38 marker (40x magnification).

**Figure 11:**
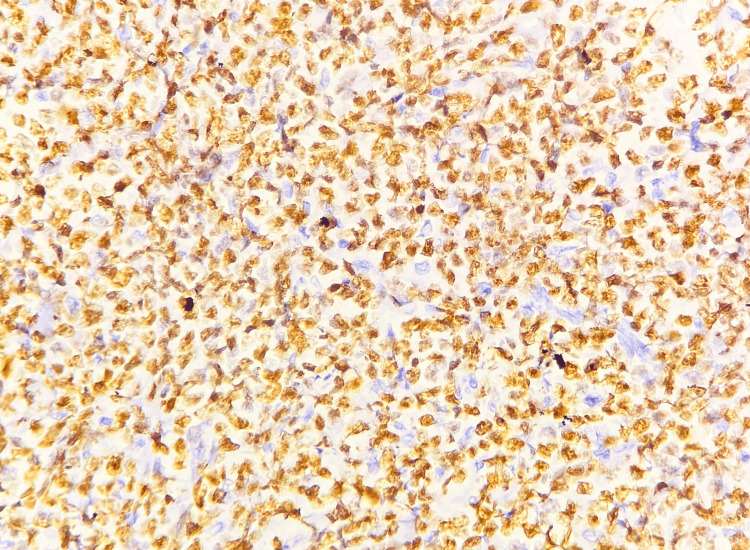
Microscopic image of IHC-stained section showing tumor cells expressing nuclear staining for Mib-1 with 90% proliferation index (40x magnification).

**Figure 12:**
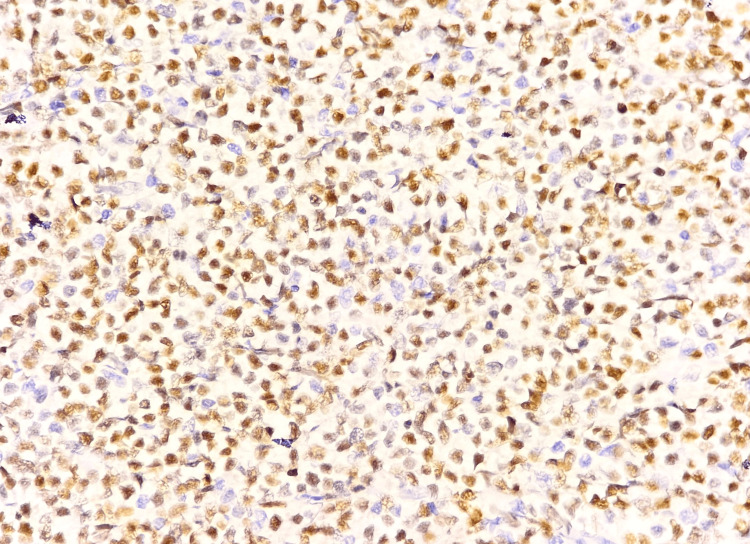
Microscopic image of IHC-stained section showing tumor cells expressing nuclear staining for c-Myc with 70% proliferation index (40x magnification).

IHC confirmed the diagnosis of DLBCL (germinal center B-cell subtype), as the tumor cells were positive for Oct-2, CD20, PAX5, BCL-6, and CD10 (Figures 6-9) and negative for CD38 (Figure 10), CD138, MUM1, and CD30. A high MIB-1 (Ki-67) proliferation index of 90% and MYC expression of 70% supported the aggressiveness of the tumor, defining it as a high-grade DLBCL (Figures 11, 12).

The immediate postoperative recovery was uneventful. The patient was then initiated on systemic chemotherapy with a modified R-CHOP (rituximab, cyclophosphamide, doxorubicin, vincristine, and prednisone) regimen, alongside continuation of antiretroviral therapy. She showed progressive clinical improvement, with resolution of GI symptoms and improved nutritional status. Follow-up imaging revealed partial regression of residual lesions, and she continues to be monitored closely in coordination with oncology and infectious disease services.

## Discussion

DLBCL is the most common subtype of NHL, accounting for approximately 30-40% of all NHL cases. It primarily affects older adults but can also be seen in immunocompromised individuals, including HIV-positive patients. The GI tract is the most frequent extranodal site of involvement, with the stomach and small intestine being the most commonly affected regions [7].

In HIV-positive individuals, lymphoma is an AIDS-defining malignancy, with DLBCL and PBL being the predominant subtypes. The risk of developing NHL in HIV patients is 10-20 times higher than in the general population, attributed to chronic immune activation, Epstein-Barr virus co-infection, and a compromised immune surveillance system. Despite the increased incidence, the introduction of combination antiretroviral therapy (cART) has improved overall survival rates by reducing immunosuppression and allowing for better tolerance of chemotherapy [8].

Clinically, GI-DLBCL often presents with nonspecific symptoms such as abdominal pain, nausea, vomiting, weight loss, and, in some cases, acute complications such as obstruction, perforation, or intussusception. Intussusception is rare in adults and is more frequently associated with malignancies, including lymphoma [9]. Imaging modalities such as CECT and endoscopic evaluation with biopsy play a crucial role in early diagnosis, but histopathological confirmation is essential for definitive diagnosis.

On histology, DLBCL is characterized by a diffuse proliferation of large atypical lymphoid cells with prominent nucleoli, high mitotic activity, and frequent apoptotic bodies [2]. The presence of plasmacytoid morphology in some cases, particularly in HIV-associated lymphomas, can lead to diagnostic confusion with PBL. IHC is commentative in differentiating these subtypes, with DLBCL expressing CD20, PAX5, BCL-6, and CD10, whereas PBL typically lacks B-cell markers and expresses plasma cell markers such as CD38, CD138, and MUM1.

The standard treatment for DLBCL is R-CHOP chemotherapy, which has significantly improved outcomes, achieving complete remission in approximately 60-70% of patients. However, in HIV-positive patients, balancing chemotherapy with antiretroviral therapy is crucial to minimize toxicity and opportunistic infections. Recent studies suggest that HIV-positive patients receiving cART alongside chemotherapy have comparable outcomes to the general population, reinforcing the importance of integrated management [6,8,10].

This case adds to the growing literature on HIV-associated GI lymphomas, emphasizing the need for early suspicion, accurate histopathological differentiation, and timely treatment. Given the overlap of clinical and imaging findings with TB in endemic regions, histopathological and IHC confirmation remain paramount in ensuring appropriate management and avoiding delays in treatment initiation.

## Conclusions

This case highlights the diagnostic challenges in HIV-positive patients presenting with GI symptoms, where TB is often the primary suspicion. However, the presence of an intestinal mass with intussusception warranted further evaluation, ultimately leading to the diagnosis of DLBCL (germinal center B-cell subtype). The case emphasizes the critical role of histopathology and IHC in differentiating lymphoma from infections in immunocompromised patients. A high index of suspicion, timely surgical intervention, and appropriate oncologic management are essential for improving patient outcomes.
